# The Emerging Era of Personalised Medicine in Advanced Colorectal Cancer

**DOI:** 10.1111/jgh.15937

**Published:** 2022-07-20

**Authors:** Claudia WK Wu, Madeleine Reid, Simon Leedham, Rashid N Lui

**Affiliations:** 1 Institute of Digestive Disease, Chinese University of Hong Kong, Hong Kong, China; 2 Division of Gastroenterology and Hepatology, Department of Medicine and Therapeutics, Prince of Wales Hospital, Hong Kong, China; 3 Translational Gastroenterology Unit, John Radcliffe hospital, University of Oxford, Oxford, United Kingdom; 4 Wellcome Centre for Human Genetics, Nuffield Department of Medicine, University of Oxford, Oxford, United Kingdom; 5 Department of Clinical Oncology, Chinese University of Hong Kong, Hong Kong, China

**Keywords:** personalised medicine, signalling pathway, Immune checkpoint Inhibition, tumour microenvironment, targeted therapy, chemotherapy, colorectal cancer

## Abstract

Colorectal cancer (CRC) is a genetically heterogeneous disease with its pathogenesis often driven by varying genetic or epigenetic alterations. This has led to a substantial number of chemoresistance and treatment failure, resulting in a high mortality rate in advanced CRC. Deep molecular analysis allowed for the discovery of key intestinal signalling pathways which impacts colonic epithelial cell fate, and the integral role of the tumour microenvironment on cancer growth and dissemination. Through transitioning pre-clinical knowledge in research into clinical practice, many potential druggable targets within these pathways have been discovered in the hopes of overcoming the roadblocks encountered in conventional therapies. A personalised approach tailoring treatment according to the histopathological and molecular features of individual tumours can hopefully translate to better patient outcome, and reduced rate of recurrence in patients with advanced CRC. Herein, the latest understanding on the molecular science behind CRC tumourigenesis, and the potential treatment targets currently in the forefront of research in this vibrant field are summarised.

## Introduction

Colorectal cancer (CRC) is the third most commonly diagnosed malignancy worldwide and has the second highest cancer mortality rate in Europe.^1^ Furthermore, the global burden of disease is predicted to increase by 60% to 2.2 million new cases a year by 2030.^2,3^ Based on positive trials of population screening, many countries have instituted population level CRC screening programmes which has improved early detection, yet 50% of patients are still diagnosed with the disease at later stages. Metastatic colorectal cancer (mCRC) currently carries a poor prognosis with an overall 5-year survival of 14%, and this statistic has not changed significantly over the past few years.^4^

**Figure 1:**
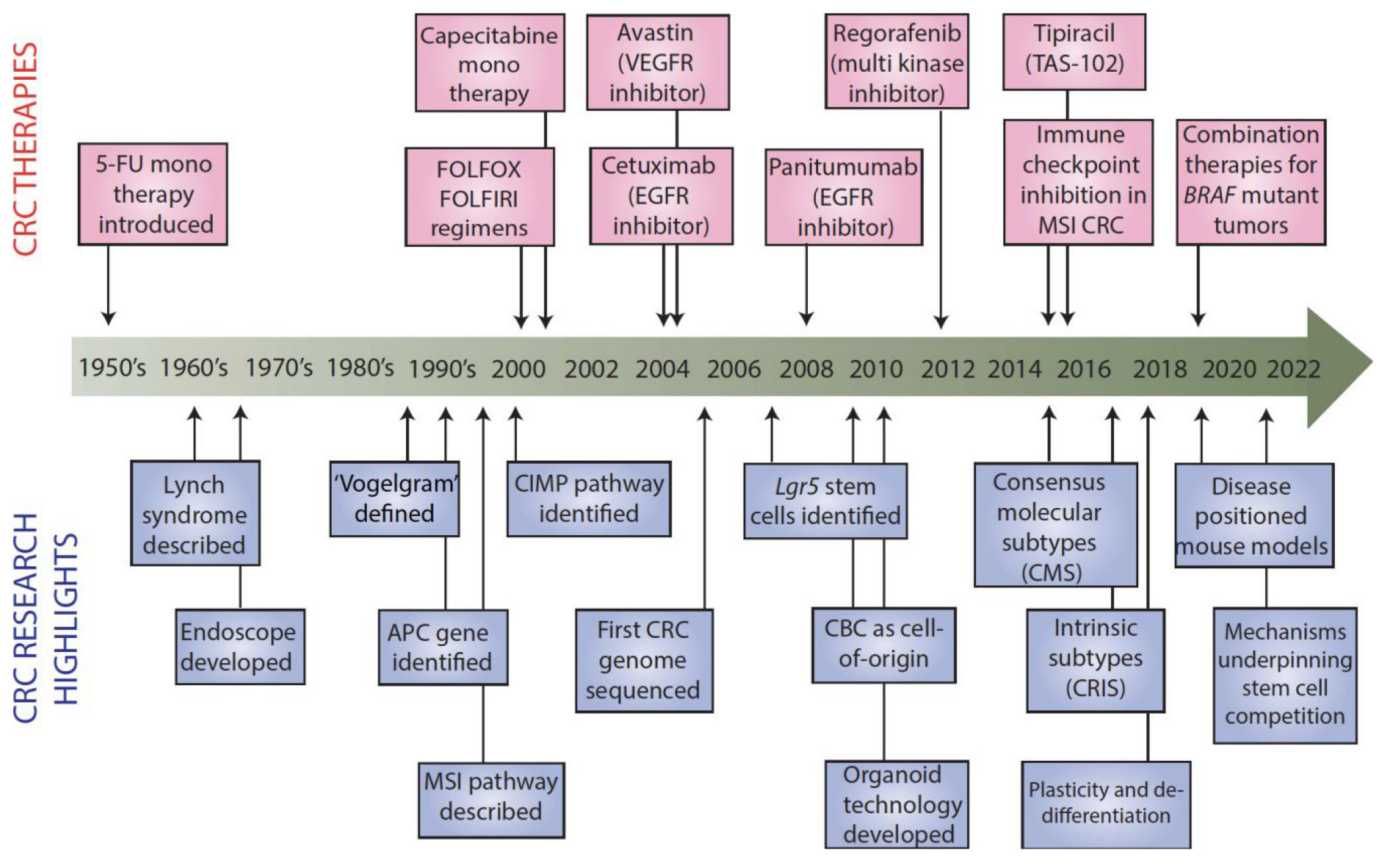
Timeline for key milestones in the history of CRC research and breakthroughs in its treatment

The one-size-fits-all model in cancer treatment has proven to be ineffective, leading to high rates of treatment failure and resistant disease.^5^ Molecular profiling of CRC has revealed high genetic heterogeneity, which includes but is not limited to chromosomal instability (CIN), microsatellite instability (MSI), and CpG island methylator phenotype (CIMP). Different subtypes of CRC carry variable prognosis and response to treatment.^6^ Recent advances in our understanding of the molecular signalling pathways that govern intestinal regeneration and homeostasis has led to promising developments for the implementation of precision medicine in CRC.^7,8^ We now have a clear understanding of the key molecular drivers that govern the pathogenesis of CRC, which, coupled with easy access to diagnostic tissue to test for actionable mutations central to disease progression has led to a new emerging paradigm in the treatment of patients with advanced disease.^9–11^ However, despite significant advances in our knowledge of tumour biology, these have not fully translated into established new treatments for all patients, with the backbone of our chemotherapeutic approach still reliant on combination cytotoxic regimens aimed predominantly at the proliferating epithelium.^12,13^ (See Figure 1)

Although there have been some successes from targeted therapies, such as that seen with the synergistic effect of BRAF-inhibitor and epithelial growth factor receptor (EGFR)-inhibitors in BRAF-mutant CRC^14^, other pathway specific therapies have failed to deliver meaningful clinical benefit in unstratified patients in Phase II and III trials e.g. NOTCH inhibitors^15^, and Regorafenib, a multi-targeted kinase.^16^ With our increasing understanding of the signalling crosstalk that regulates intestinal cell fate in health and disease, and the role of the tumour microenvironment (TME), therapeutic exploitation of inter-compartmental signalling in the malignant epithelium could represent an important new drug paradigm in CRC, as demonstrated by the effect of immune checkpoint inhibition in microsatellite unstable CRC.^17^

In this review, we have subdivided these key signalling pathways into those that regulate intestinal epithelial or cancer cell fate directly, and those that act indirectly by harnessing the power of the TME. For each, we will assess the impact of signalling on cancer cell fate and discuss some potential therapeutic opportunities that arise from successful pathway manipulation.

### 1 Regulating intestinal epithelial cell fate

The gastrointestinal stem cells are responsible for intestinal development and maintaining tissue homeostasis amidst the high turnover of intestinal epithelium in order to withstand the stress of harsh luminal contents and inflammatory processes encountered throughout our lives.^18^ Stem cells are defined functionally, through self renewal and multipotency and are thought to be the cell-of-origin of intestinal tumourigenesis.^7^ Regulation of these intestinal stem cells and epithelial cell fate, occurs through the interaction between two integral signalling pathways: the Wingless and int1 (Wnt), and bone morphogenetic protein (BMP) pathways.^19^ Colonic cell growth and apoptosis are also regulated by the complex yet pivotal epidermal growth factor (EGF) pathway.^20,21^ The balance of these pathways is crucial to the tight regulation and control of cell fate in the epithelium and critical gene mutations that disrupt these signalling cascades can tip this delicate balance and initiate tumourigenesis.^22^ Here, we discuss each of these pathways, explore their role in tumourigenesis, and investigate key components that represent potential targets for therapeutic interventions.

#### Wnt pathway

The Wnt signalling pathway has unquestionable importance for its role in embryogenesis and tissue regeneration.^23,24^ Wnt signalling is upregulated during tissue repair and development.^25^

Although integral to normal physiological cellular proliferation, hyperactivation of the Wnt pathway has a substantial role, and is almost universal in all types of CRC.^26^ It is thought to preserve cancer stemness, protect tumour cells from immunosurveillance, foster secondary tumour growth and drive cancer metastasis.^27–30^

**Figure 2:**
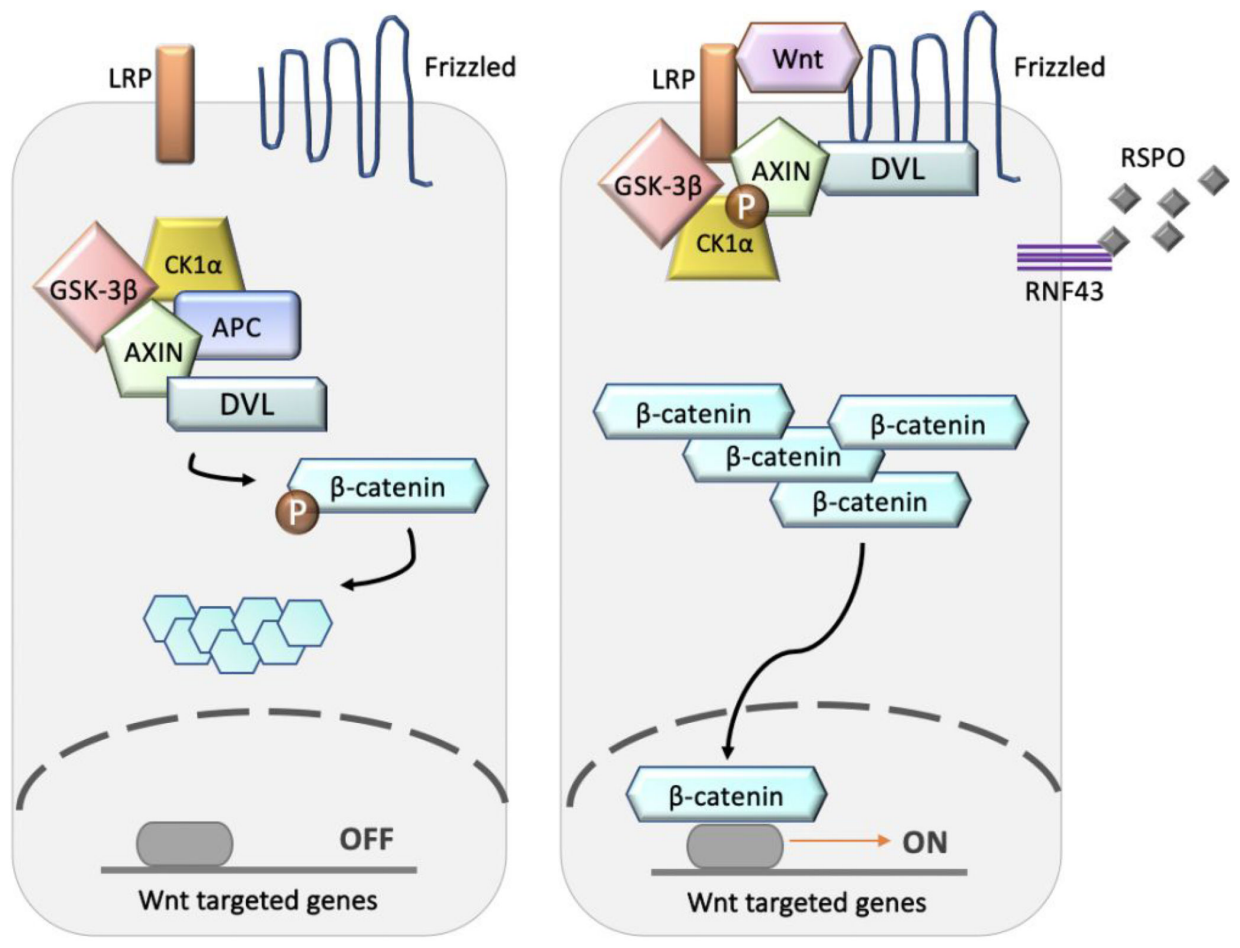
Schematic representation of the Wnt signalling pathway.

The Wnt signalling pathway (See Figure 2) is either in “on” or “off” mode in normal colonic crypts.^31^ In the absence of Wnt ligand (“off” mode), protein β-catenin is bound by a destruction complex containing adenomatous polyposis coli (APC), AXIN and protein kinases CK1α and GSK-3β. The destruction complex leads to degradation of β-catenin. Without β-catenin, a master transcription regulator of the Wnt pathway, gene expression is inhibited. In the presence of Wnt ligand (“on” mode), the signalling cascade begins when a Wnt ligand is secreted from stromal cells in the stem cell niche, activated by porcupine protein. The Wnt ligand binds to the transmembrane receptor protein Frizzled (FZD), causing activation of FZD and lipoprotein receptor-related protein (LRP). The LRP receptors are then phosphorylated by CK1α and GSK3β, which recruits Dishevelled and AXIN proteins to the cytoplasmic tail of LRP. This inhibits the formation of the destruction complex, enabling β-catenin accumulation in the cytoplasm, which then moves into the nucleus, thus allowing the targeted gene to be expressed. R-spondin (RSPO) ligands are also produced by stromal cells to inhibit ring finger protein 43 (RNF43), which normally degrades FZD. This further augments Wnt signalling.^32^

Multiple driver mutations within this pathway are selected in CRC tumourigenesis. Recent findings have suggested that mutations within the Wnt pathway can be divided into either ligand-independent or ligand-dependent mutations that can give rise to distinct tumour subgroups with non-overlapping epigenetics, molecular pathogenesis, Consensus Molecular Subtypes (CMS), and clinical characteristics.

Ligand-independent mutations drive downstream activation of the Wnt cascade, through mutational inactivation of APC, the key tumour suppressor gene (80%), or gain-of-function CTNNB1 mutations (<5%) which also result in ligand-independent mutations. Both result in constitutive activation of the pathway, independently of receptor ligand binding. Ligand-dependent mutations, on the other hand, activate the R-SPO-LGR-RNF43 axis and result in upregulation of Wnt frizzled receptors, rendering cells exquisitely sensitive to any wnt ligand stimulation. Loss of function mutations in RNF43 or gain of function R-SPO fusion mutations activate wnt, but remain dependent on the presence of activating wnt ligand. The mutual exclusivity of these mutations within the Wnt pathway make them potentially druggable targets for different CRC subtypes, some of which are described below.

Ligand-independent mutations. Ligand dependent wnt mutations are difficult to drug as the wnt pathway is constitutively activated at the signal transduction level. Tankyrase enzyme inhibitors have been shown to induce formation of β-catenin destruction complexes, which promotes the degradation of β-catenin. It can also enhance AXIN activity, which is normally a rate limiting factor for the stabilisation of these destruction complexes. AXIN2 is a negative regulator of the “on’ mode Wnt pathway, thus pursuing ways to enhance the level of AXIN2 is another promising avenue to explore. TNIK inhibitors target Traf and Nck-interacting protein kinase, which regulates the β-catenin transcriptional complex responsible for activating the expression of the targeted gene. BCL9 and BCL9L proteins (BCL9/9L) enhance the transcriptional output of β-catenin leading to high Wnt signalling, which is crucial for maintaining CRC steminess. Loss of BCL9/9L accelerates differentiation and delayed tumour growth in APC or KRAS tumours. The development of BCL9/9L inhibitors may provide an avenue for downstream β-catenin inhibition in these tumour subtypes.^33^ Several other small molecules have also come to light in recent years, including BBI608 (a STAT3 inhibitor) and CBP/ β-catenin antagonist, etc.^34–36^

Although targeting the Wnt pathway seems attractive, therapeutic targets to this pathway have shown to induce significant side effects including diarrhoea, vomiting, kidney injuries, bone and intestinal toxicity, etc.^37^ Tankyrase inhibitor has been found to induce dose dependent reversible intestinal toxicity, with risk of necrotizing and ulcerative enteritis seen in mice.^38,39^ Uncontrolled Wnt signalling can cause kidney fibrosis in patients with chronic kidney disease.^40^ Significant bone toxicity, including loss of bone density and increased risk of fractures, has limited some drugs from moving forward in clinical trials.^41,42^ Nevertheless some studies have suggested that the coadministration of bisphosphonate therapy can potentially overcome the risk of bone toxicity.^40,43^ Careful balancing between achieving therapeutic effects and minimising unwanted “off-target” side effects is needed in the development of these targeted therapies.

Ligand-dependent mutations. Ligand-dependent mutations in R-spondin and RNF43 result in disruptions of the RSPO axis.^44^ RNF43 mutations are frequently truncated frameshift mutations with tandem repeats of microsatellites.^45^ Thus, these mutations often occur in sporadic MSI colorectal tumours, with MLH1 mismatch repair gene being the most frequently associated mutation, of right colonic distribution, and CMS1 tumour molecular subtype.^46–48^ Fifty percent of sessile serrated lesions are thought to have RNF43 mutations. Ligand-dependent tumours are rich in mucins,^49^ reflected by the fact that RNF43 mutations are seen in 34.5% of genetic mutations responsible for mucinous adenocarcinoma, which are also more commonly associated with MSI.^50,51^ R-spondin gene fusion mutations lead to stromal overexpression and upregulation of the Wnt pathway in 10% of CRC.^52^ RSPO fusion mutations can induce ectopic crypt formation and are often documented genetic alterations arising from traditional serrated adenomas as well.^53^

Increasing evidence suggests that ligand-independent mutations occur mutually exclusively from ligand-dependent mutations, giving rise to distinct tumour subtypes and clinical characteristics.^54^ These tumours can be stratified using simple molecular markers. Furthermore, with ligand-dependent mutations the canonical Wnt pathway remains intact, so reduction in ligand availability causes an appropriate reduction in pathway activity ^55^ Multiple novel molecular targets have been studied to modulate the ligand-dependent pathway of the aberrant Wnt cascade in different types of cancer, also showing promise within CRC treatment.^56^

Porcupine inhibitors target the porcupine protein, which is essential for the secretion of Wnt ligands.^57^ Several porcupine inhibitors have entered Phase I clinical trials and have been shown to decrease β-catenin and Wnt3 levels, downregulating Wnt signalling and stimulating cancer cell dormancy.^58,59^ They have shown promise in the treatment of different types of cancer, including CRC, hepatocellular cancer, pancreatic cancer, etc.^57^ A recombinant fusion protein, Ipafricept, which acts as a Wnt/FZD antagonist, competes with FZD receptors by binding directly to Wnt ligands.^26^ Human phase I studies revealed compelling results against solid organ tumours, showing a selective reduction in cancer stem cells.^60^ Yet bone toxicity at therapeutic doses of ipafricept has limited the drug from proceeding further in clinical trials with treatment of ovarian cancer.^61^ RSPO acts as a powerful stem cell growth factor by potentiating the Wnt pathway.^44^ A clinical trial on anti-RSPO molecules in combination with taxane treatment has demonstrated efficacy in reducing cell proliferation and increasing the number of differentiated cells in patient-derived xenograft tumours harbouring RSPO3 fusions.^62^

#### BMP pathway

The BMP pathway counteracts the pro-stem and pro-proliferative effects of the Wnt pathway, regulating cell differentiation and apoptosis in intestinal homeostasis.^63,64^ BMP ligands are members of the transforming growth factor-β (TGF-β) signalling family.^65^ They have crucial roles during embryogenesis, mainly in mesoderm formation, cardiac and bone development.^66,67^ Later on, they were found to be a ubiquitous molecule which plays a vital role in almost all organ systems, and are involved in cellular growth, apoptosis and differentiation of specialised cells within the intestinal epithelium.^68,69^

BMP signalling pathway is initiated when BMP ligands bind to the transmembrane tyrosine kinase receptors BMP type II receptors (BMPRII), which then recruits and phosphorylates BMP type I receptors (BMPRI). This complex then initiates an intracellular signalling cascade by phosphorylation of the intracellular proteins SMAD1, 5, and 8. A transcriptional regulatory complex is formed after binding with SMAD4. This complex moves into the nucleus and leads to the activation of the BMP pathway target genes.^70^

Interactions between the BMP and Wnt signalling pathways are critical to crypt-villus homeostasis. Within the normal intestine, Wnt with predominantly progenitor properties are produced at the crypt, whereas BMPs that promote differentiation are produced at the top of the villus.^71^ Stromal myofibroblasts at the crypt base also produce BMP antagonists, such as Gremlin 1 (Grem1), preventing the stem cell niche from being exposed to BMPs.^72,73^ Germline mutations disrupting the BMP pathway are responsible for two hereditary polyposis syndromes, hereditary mixed polyposis syndrome (HMPS) (through mutational upregulation of the secreted BMP antagonist, Grem1) ^74^ and juvenile polyposis syndrome (JPS) (through mutational inactivation of BMPR1A or SMAD4). Furthermore, BMP is the predominant pathway affected by known common predisposition variants in CRC.^73,75–77^ Upregulation of SMAD1 is seen in p53 mutated tumours acting to stabilise p53 and suppresses oncogenesis.^78^

In recent years, Grem1 has shown to be a promising target for therapy.^79^ As a BMP antagonist, Grem1 downregulates BMP activity, promotes angiogenesis, reduces cell apoptosis and maintains cell stemness especially within the stem cell niche.^80^ Administration of recombinant Grem1 has demonstrated ability to stimulate migration and invasion of endothelial cells in lung tumours. Grem1 is also overexpressed in the desmoplastic invasion front of CRC, a zone where many cancer metastases are speculated to originate. The pathogenesis behind HMPS stems from a large duplication just upstream of the Grem1 gene, resulting in Grem1 overexpression, and the development of polyps of mixed morphologies from a young age.^81^ At elevated levels, Grem1 has been linked to tumour progression and risk of metastatic dissemination in several types of cancers.^79,82^ Therapeutic targeting of Grem1 may prove to be a pivotal therapeutic avenue that can alter the fate of cancer driven by the BMP pathway, though future studies are needed to elucidate this.^83^

#### EGF pathway

EGFR is a transmembrane tyrosine kinase receptor that belongs in the ErbB cell membrane receptor family.^84^ The EGFR signalling cascade is one of the most important pathways responsible for regulation of cell proliferation, angiogenesis, migration, and inhibition of apoptosis.^84,85^ It regulates key cellular events that drive the progression of not only CRC, but other neoplasms, including lung, pancreatic, breast cancer, etc.^86–88^

**Figure 3:**
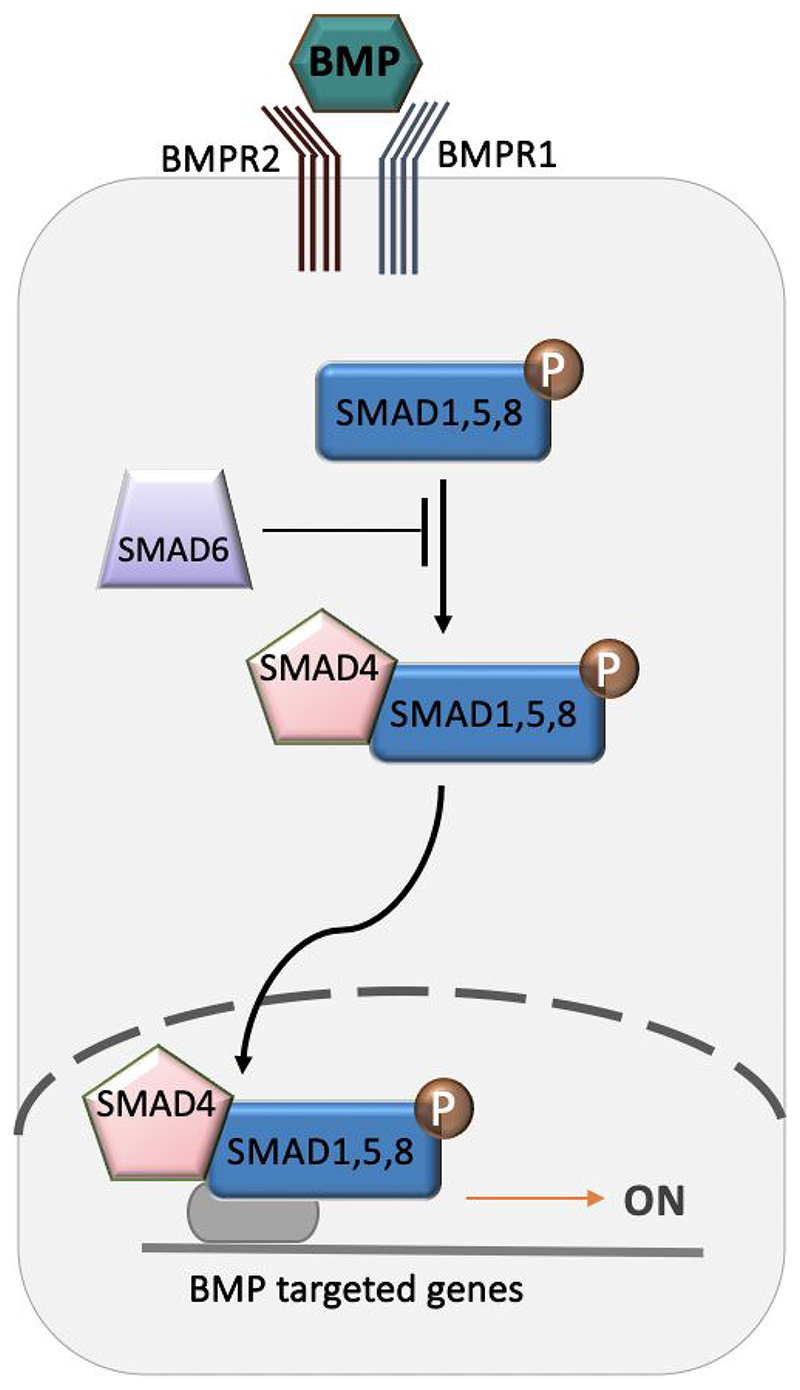
Schematic representation of the BMP signalling pathway.

The EGFR pathway begins with ligand activation of EGFR (Figure 3).^89^ This causes conformational change in the extracellular domain of the EGFR and leads to dimerization with another EGFR. EGFR dimerization induces phosphorylation of the carboxyl terminal of tyrosine kinase. This serves as a docking site for proteins containing Src homology2 (SH2) or phosphotyrosine binding (PTB) domains. The binding to this receptor triggers activation of multiple signal transduction cascades important for cell growth and survival.^90^

Two of the main pathways activated by EGFR are mitogen-activated protein kinase (MAPK) and phosphoinositide 3-kinase (PI3K)/ protein kinase B (AKT) pathway.^91,92^ The activation of the MAPK pathway starts by the binding of growth factor receptor bound protein 2 (Grb2) and Sos protein to EGFR C-terminal tyrosine residue.^93^ Through a cascade of protein kinase activation, which consists of RAS, RAF, MEK and ERK, the activated MAPK enters the cell nucleus stimulating transcription factors to express genes responsible for cell proliferation.^94^ The PI3K/AKT pathway, on the other hand, is initiated by the binding of p85 subunit of PI3K to the EFGR receptor, leading to dimerization of EGFR with another member of the ErbB family, HER3.^95^ The activation of the PI3K/AKT pathway regulates cell migration and apoptosis.^96,97^

Altered gene activity in these pathways leads to uncontrolled tumour proliferation and apoptosis. EGFR overexpression in CRC is typically thought to be associated with poor survival and tumour progression.^98^ Anti-EGFR agents, such as cetuximab and panitumumab have had an established role in the treatment of mCRC.^99,100^ Cetuximab is an IgG1 monoclonal antibody that competitively binds to EGFR, inhibiting the phosphorylation of EGFR tyrosine kinase.^100^ Its use as a single agent or in combined therapy, such as the FOLFOX regimen, has been studied in many trials.^101–103^ Panitumumab is a recombinant IgG2 anti-EGFR monoclonal antibody that is mainly used as combined therapy with other chemotherapy agents for wild-type KRAS tumours.^104,105^ The use of these anti-EGFR agents, however, are limited by skin toxicity and venous thromboembolic events.^106^

The MAPK pathway also offers many potential targets for therapy. The MEK inhibitor is a promising immunotherapy that inhibits the RAS/RAF/MEK/ERK signalling pathway, leading to reduced cell proliferation and induce cell death.^107,108^ It is currently under investigation for several cancer types, such as non-small cell lung cancer, and breast cancer.^109,110^ Trametinib and cobimetinib are two MEK inhibitors that have been Food and Drug Administration (FDA) approved for the treatment of melanoma.^111^ Nevertheless, specific to their use as CRC therapy, a phase III randomised control trial on cobimetinib combined with immune checkpoint inhibitor atezolizumab (IMblaze370) failed to meet the primary endpoint to improve overall survival in patients with metastatic CRC.^112^ Several other MEK inhibitors are currently undergoing phase I/II clinical trials.^113–115^

Resistance to therapy is a major obstacle to anti-EGFR treatment.^116^ It was discovered that different gene alterations within the EGFR pathway can impact on prognosis and predict responsiveness to anti-EGFR therapy.^117,118^ There are complex innate and acquired factors that may contribute to drug resistance.^119^ Proto-oncogenes RAS and RAF play important roles in transducing EGFR signalling.^120^ Mutations within the RAS family, including KRAS and NRAS are associated with resistance to anti-EGFR therapy.^121^ Mutations of BRAF is the most common mutation in the RAF family occurring in 10-15% of CRC.^122,123^ It results in phosphorylation of MEK and ERK leading to increased activation of the MAPK pathway and confers resistance to anti-EGFR therapy.^124^ Molecular profiling prior to treatment initiation can allow better tailoring of therapy, with anti-EGFR drugs best used on patients with BRAF, KRAS and MRAS wild-type CRC.^125,126^ Recently, the BEACON study provided a crucial breakthrough using the combination of a BRAF inhibitor with an EGFR inhibitor which showed improved overall survival in BRAF-mutant advanced CRC.^127^ This was also the first instance where a chemotherapy-free regimen was shown to be effective in this CRC subgroup. Mutations in the PI3K/AKT pathway, and low expression of PTEN and TP53 levels are also predictive of poor anti-EGFR efficacy.^128–130^ PI3K inhibitors and mTOR inhibitors are the two main targeted therapies directed against the PI3K/AKT pathway that have been used in renal cell carcinoma or in breast cancer, yet their use in CRC has not been well established, requiring further evaluation in clinical trials.^131–134^

To date, many potential agents aimed at overcoming anti-EGFR drug resistance are entering the early phases of clinical trials, making this a hot topic for research. Combined targeted therapy to overcome resistance mechanisms may be the way forward to improve survival of late stage CRC.^118,135^

#### 2 Immune checkpoint inhibition therapy in MSI/dMMR cancers

Immune checkpoint inhibition therapy unleashes the power of the endogenous immune system to kill mutated cancer cells and has delivered the biggest oncological breakthrough in recent years, revolutionising the management of some solid tumours (such as melanoma and lung cancer). It is most effective in cancers with a high epithelial mutation burden through significant neoantigen formation.

In colorectal cancer, MSI is a well described subset of phenotypes with an abnormally high frequency of intragenic mutations of short DNA tandem repeat sequences, termed microsatellites, due to the loss or epigenetic silencing of DNA mismatch repair (MMR) activity or a defect in this system. This accounts for about 15% of CRCs, including patients with the hereditary cancer syndrome of Lynch syndrome, and carries distinct features compared to other tumour types.^136–138^ Approximately 80% of cases of sporadic mismatch-repair deficiency (dMMR) in CRC are due to MLH1 gene promoter methylation, and over 70% of hereditary dMMR incidents are coupled with MLH1 and MSH2 germline mutations.^138–140^ Only approximately 4% of mCRCs are deemed as dMMR tumours, whereby upwards of 30% of the microsatellite marker panel are mutated. Hence, MMR DNA deficiency is also known as microsatellite-instability high (MSI-H).^138,141^ There is increasing literature suggesting that MSI-H/dMMR tumours show a reduced response to chemotherapy-based treatment, thus an alternative strategy for this subset of CRCs is crucial.^141,142^

MSI tumours have unique immune profiles, which lends evidence to different immune escape mechanisms, and highlights the potential role for individualised immunotherapeutic strategies.^143^ The high mutational burden of MSI CRC tumours causes neoantigens to be loaded onto antigen-presenting cells, which are subsequently flagged by T cells.^144,145^ These frameshift mutations cause changes in protein structure and can lead to antigenic epitopes, enhancing the immunogenic nature of these cancer subtypes.^146^ Patients with dMMR/MSI-H tumours tend to have good prognosis in cases of early stage CRC, but this becomes less favourable in the small number of MSI patients with metastasis (4%).^147–149^ These cancers are characterised by a tumour-infiltrating lymphocyte-rich microenvironment, as well as upregulated immune checkpoint inhibitors (ICIs) that act to protect MSI tumour cells from their unfavourable immune microenvironment, through depletion of cytotoxic T cells. Upon powerful activation of immune cells, a feedback expression is initiated consisting of immune checkpoint blockade ligands and receptors, including programmed cell death-1 (PD-1) and programmed cell death ligand 1 (PD-L1).^143,150^

Evidencing the efficacy of targeting ICIs within CRC, a phase I clinical study focusing on MSI/dMMR metastatic CRC (mCRC) showed a long lasting complete response of a patient following anti-PD1 ICI treatment.^151^ Following this, several non-randomised phase II studies have shown exciting data, with response rates that varied between 30-60%, as well as displaying lasting clinical outcomes.^150,152,153^ As such, ICIs have become a recent addition to CRC therapeutic options, consisting of monoclonal antibodies (mAbs) that target inhibitors of T cell receptor (TCR) activation, especially PD-1, PD-L1, and cytotoxic T lymphocyte-associated antigen-4 (CTLA-4). Pembrolizumab and nivolumab, both mAb ICIs that target PD-1, have shown promising, durable anti-tumour results in MSI-H/dMMR CRC patients, and have led to FDA approval for both drugs for this subset of patients within the last few years to be used as a salvage therapy.^154^

A recent randomised phase III trial presented further encouraging data for biomarker-driven studies that aim to target MSI-H/dMMR CRCs. Pembrolizumab showed improved progression-free survival rates, and reduced treatment-related adverse effects when compared to chemotherapy across various patient subgroups. These affirmative results have led to FDA approval of pembrolizumab as first line therapy for advanced CRC patients with MSI-H/dMMR tumours. This also holds important clinical significance for regulatory agencies that are still sceptical of ICI drug approval based on single-group studies.^139,155^ Ipilimumab, a CTLA-4 inhibiting fully humanised monoclonal antibody, has also recently been approved by the FDA for combination therapy with nivolumab in patients with MSI-H mCRC who have previously had chemotherapy.^156^

It should be noted however, that despite the high response rates and durable clinical benefit seen with ICIs, up to 50% of MSI/dMMR mCRC tumours have been reported to display primary or secondary resistance to immunotherapy and will eventually progress. This resistance is gradual, suggesting that changes in the TME and the tumour genome are occurring, which result in this acquired ICI resistance (9, 21, 24).^139,143,157^

MSI-H tumours carry a high and variable tumour mutational burden (TMB), which has recently been shown to be a predictor of ICI treatment response. This may be responsible for the observed heterogeneous responses to anti-PD-1 therapies, thus highlighting a role for TMB scoring when making decisions regarding the best line of treatment. With the progress of these drugs in front-line mCRC treatment and further encouraging immunotherapy data emerging, the identification of extra biomarkers to identify such heterogeneous subtypes, is paramount to help guide effective immunotherapy for dMMR/MSI-H CRC.^158^ It is also important to consider that a significant proportion of treatment resistant tumours have been mistakenly diagnosed as MSI-H/dMMR. Consequently, when assessing tumour progression in the context of ICIs, the possibility of a misdiagnosis should be determined.^159^ Despite these obstacles, pembrolizumab remains the current favoured treatment option for MSI-H/dMMR advanced CRC due to its durable response rates, encouraging safety reports and increased quality of life.^155^

### 3 Role of the tumour microenvironment within CRC

Currently the majority of chemotherapies predominantly target the proliferating cancer epithelium alone, however, increasing evidence shows a key role for the TME in regulating and controlling epithelial cell fate. Solid tumours are complex ecosystems with the mutated epithelial embedded within, and interacting with desmoplastic stroma, a dense extracellular matrix, neovascularized endothelium and a constellation of immune cells. Intercompartmental crosstalk through soluble morphogens, cytokines and metabolites regulates co-evolution of the cancer cell and the TME. Effectively harnessing the power of these interactions and exploiting the power of the TME in controlling cancer cell behaviour could lead to a new therapeutic paradigm for solid tumours.

#### Anti-TGF-β strategies in colorectal cancer stromal cells

There is a distinct phenotype of stromal cells that exists within the TME which act to support tumour cell growth, survival, and metastasis. Though these are not automatically tasked with tumour promotion, over time, they are influenced by the dynamic interaction they have with tumour cells, undergoing spatial and temporal changes in stromal cell architecture. This complex stromal system can therefore act to enhance CRC cell growth and survival.^160,161^ In mouse models, inflamed stroma has been shown to enhance the evolution of colonic adenomas to adenocarcinomas, while in humans, stromal rich CRC has been linked to poor prognosis, lower therapy response rates and a predilection for metastasis.^160,162^

The TGF-β signalling pathway is a pleiotropic morphogen, which plays a key intercompartmental, homeostatic role in control of several developmental cell fate decisions and is frequently co-opted and corrupted in cancer. Convolutedly, it presents itself as a context-dependent paradox regarding its function. In normal tissue, it can act to block epithelial growth, while in a cancerous environment, it increases tumour cell progression.^163^ Mutational inactivation of TGF-β signalling via tumour-stromal interactions is a crucial player in CRC progression, and alterations in this pathway have been shown to affect 40-50% of all CRCs, which renders the epithelium resistant to the cytostatic and pro-differentiation effects of the TGF-β ligand. However, non-epithelial cells remain responsive, and TGF-β acts as a master regulator of the TME, inducing recruitment of circulating mesenchymal stem cells (MSC), induction of cancer-associated fibroblast (CAF) differentiation, activation of endogenous fibroblasts, promotion of angiogenesis, initiation of epithelial-to-mesenchymal transitions (EMT) and the establishment of an immunosuppressive microenvironment.^163–165^ This TME landscaping contributes to matrix remodelling and desmoplastic reaction with resultant changes in mechanotransduction, linked to tumour aggression, treatment failure, chemo- and radiotherapy resistance, and poor survival in many malignancies including glioma, pancreatic, breast, colorectal and prostate cancers.^166–168^ Use of this pathway in CRC therapeutic studies has generally been avoided due to concerns over possible accidental tumour promotion. However, recent progress in molecular stratification and animal models shows promise for increasing our understanding of differential tissue compartmental responses to TGF-β, which may help inform precision targeting of the TGF-β pathway in the near future.^169^

TGF-β signalling is also known to activate a variety of tumour stroma cell types, however CAFs are accepted as being the predominant contributors when linking stromal TGF-β with reduced therapeutic outcome in CRC. Persistent inflammation in CRC causes sustained fibroblast activation, which further exacerbates TGF-β production and results in a perpetual and pathogenic wound-healing programme.^170,171^ TGF activated CAFs secrete an abundance of TGFβ-induced factors that further contribute to CRC cell proliferation. Their ability to secrete interleukin-11 (IL-11) for instance, increases initiation of metastasis in cancer cells. Encouragingly, upon inhibition of IL-11 through addition of a potent IL-11 agonist, both CRC cell proliferation and invasive capacity were seen to decrease.^172,173^

Increased stromal TGF-β expression in CRC and marked extracellular matrix (ECM) deposition has been shown to be a prevalent component of poor prognosis and deficient immunotherapy responses, likely due to TGF-β in CAFs enhancing the tumour-initiating ability of CRC cells, and thus their metastatic potential.^169^ An alternative strategy to target TME elements for destruction is to induce the repolarization of stromal cells into a non-tumour progressive condition. This may allow for lower toxicity rates compared to destructive therapies, and would provide an effective method to combine with other treatment options. As TGF-β is associated with stromal pro-tumourigenic activity within CRC, it is logical to assume that this could provide a powerful therapeutic strategy if this can be successfully inhibited.^174^

Pharmacological inhibition of the TGF-β signalling pathway in the TME has been shown to favour this approach, as metastasis formation was prevented in patient-derived tumour organoids. This supports the role of TGF-β dependent signalling in stromal cells to control the metastatic process.^162^ Various studies have demonstrated that TGF-β neutralisation causes decreases in both ECM density and myofibroblastic CAFs, which are correlated with hindered anti-tumour immune responses. Moreover, it seems to permit stromal remodelling in the TME, with concomitant significant improved efficacy of immune checkpoint inhibition.^175–177^ A separate study has highlighted a pro-tumourigenic and angiogenesis inducing function for TGFBI, an TGFβ-inducible ECM protein, when expressed in mCRCs. Anti-TGFBI targeting therapies may therefore prove to be an effective strategy, possibly in combination with other treatments.^172^ Furthermore, using combined inhibition of both TGF-β and PDL-1 has been shown to abrogate primary and metastatic tumour immune evasion in murine models of CRC and improved their survival. This demonstrates the exciting possibility of targeting TME-specific pathways in patients with advanced CRC.^178^ Recent clinical data has also demonstrated a positive response for combined treatment with a TGF-β receptor 1 inhibitor (vactosertib) plus pembrolizumab in patients with last line metastatic MSS CRC.^179^

Interestingly, ionising radiation has been shown to cause a dose dependent increase in TGF-β ligand availability, predominantly through its release from its extracellular bound latent form. TGF-β is a master regulator of radiotherapy-induced anti-tumour immunity and inhibition of TGF-β with a pan-TGFb antibody has been shown to enhance immune responses, which were further enhanced by the addition of PD-1 inhibition. Thus, combining TGF-β neutralisation with local radiation therapy may provide a novel personalised therapeutic option for patients with rectal cancer undergoing combination chemo-radiotherapy.^180,181^

Of note, a TGFβ-dependent stromal subset was recently characterised within MSI-H/dMMR CRC, exhibiting an increase in both angiogenesis and tumour neovasculature, as well as abnormal control of ECM remodelling. As such, these results are in line with the established tumour-promoting function of a TGFβ-activated TME with an abundant CAF population. This is also thought to be associated with the risk of ICI resistance, hence, a dual inhibition has been suggested of both TGF-β and immune checkpoints in this subset of CRC patients.^182^

#### Therapeutic targeting of VEGF expression in endothelial cells

Within the TME, endothelial cells act to communicate between tumour cells and the surrounding areas through the generation of new vascular networks, or by the modification of those that are already present. In doing so, they support a supply of oxygen and nutrients to tumours.^183^ Without this, tumours fail to grow beyond a couple of millimetres in size, thus neovascularization has become a natural target for cancer treatment, including CRC.^184,185^ In the context of mCRC, antiangiogenic agents are currently considered to be amidst the most successful options, and are often recommended for use in combination therapies.^186^

The molecular process underlying tumour angiogenesis is acutely intricate and dynamic. Tumour cells produce a variety of blood vessel promoting factors, including vascular endothelial growth factor (VEGF), chemokines, angiopoietins, and basic fibroblast growth factor.^187^ Arguably the most influential of these are VEGFs and their receptor family (VEGF receptor, VEGFR), which play a vital part in the pathology of angiogenesis, through the stimulation of endothelial cell proliferation and migration, influencing the permeability of blood vessels, and altering both the function and morphology of these.^185^ High VEGF expression levels are correlated with tumour stage and progression, linking them to CRC cases with a poor prognosis.^188^ Various studies have implied an important role for VEGF overexpression in cancer. For example, two different splicing isoforms (VEGF121 and VEGF165) have been shown to be differentially expressed in CRC. VEGF165 in particular demonstrated increased tumour expansion through smooth muscle cell recruitment and vessel maturation.^189^

The role that the VEGF-VEGFR axis plays with regard to tumour pathogenesis and angiogenesis has naturally driven research into developing therapeutic agents that target this. To date, several anti-VEGF/VEGFR drugs have proven effective at inhibiting tumour growth, metastasis, and angiogenesis in CRC and other cancers.^185,190,191^

Bevacizumab was the first available anti-angiogenic therapy, and has been characterised extensively for its anti-VEGF effects. It is a recombinant humanised monoclonal IgG1 antibody, which targets the TME through binding to VEGF-A isoforms, neutralising these, preventing binding with VEGF tyrosine kinase receptors and VEGF-A incorporation. Subsequently, cell viability is inhibited, and both apoptosis and autophagy are induced, thus inhibiting tumour progression and metastatic spread.^145,192–194^ Tumour hypoxia, known to play an important role in anti-angiogenic drug resistance, has also been observed under bevacizumab treatment.^195^ A recent study showed that following inhibition of autophagy, bevacizumab-induced apoptosis was significantly increased, which was also associated with enhancing the inhibition of proliferation in vitro. This suggests a possible role for combining autophagy inhibitors with bevacizumab within CRC treatment.^196^ Moreover, bevacizumab is thought to work best in combination with chemotherapy and has proven to increase survival rates in patients with mCRC, as well as prolonged progression-free periods.^197,198^

Aside from bevacizumab, a variety of other anti-angiogenic factors have been studied. For instance, Aflibercept, a humanised recombinant fusion protein, consists of a VEGF-binding segment which fuses to the Fc part of human immunoglobulin G1^199^. In combination with FOLFIRI chemotherapy, Aflibercept has also shown increased survival rates in patients with mCRC.^200^ Other approved treatments targeting angiogenesis in mCRC include ramucirumab, a VEGFR-2 extracellular domain-binding, fully humanised immunoglobulin G1 monoclonal antibody, that acts to inhibit VEGF ligand binding.^201^ An alternative option to these is regorafenib, which acts to block angiogenic tyrosine kinases (VEGFR-1 and VEGFR-3) amongst other pathways.^16^

Despite anti-VEGF therapy for mCRC becoming widely adopted, this still presents several challenges including anti-angiogenic therapy resistance, which is thought to be largely correlated with tumour endothelial cell (TEC) heterogeneity.^202^ Preclinical studies have shown that in these patients, tumours are able to cultivate compensatory mechanisms, which consequently restore angiogenic density and therefore, cancer progression. It has been suggested that this phenomenon is linked to an upregulation of pro-angiogenic components, triggered by a state of hypoxia.^203^ However, the complete mechanism of resistance to anti-VEGF therapy is still yet to be completely understood, and no biomarkers to date have yet been developed to successfully predict patient responses to anti-angiogenic treatment.^204^ Thus, further studies are essential to further understand TEC interactions with VEGF and the TME, which is a fertile field to further improve the therapeutic options available for advanced CRC.

## Conclusion

Increasing availability of cancer genome sequencing has expanded the horizon of our understanding behind the mechanisms underlying CRC carcinogenesis. Through pathway manipulation, personalised oncology with targeted therapies have the potential of producing substantial improvement in the prognosis of advanced CRC. The Wnt, BMP and EGFR signalling cascade are some of the molecular pathways that have gained attention in the field of oncology in recent years. As our understanding deepens regarding the intricate ties between the complex ecosystem of the TME and their interaction with tumour cells, exploring new small molecules to more effectively target the TME front has also gained momentum. The advent of immune checkpoint inhibitor therapy has led to a paradigm shift in how we can treat these tumours and its interaction with a whole plethora of unique molecular landscapes is still awaiting further exploration. Additional in-depth research and clinical trials of small molecules targeting these crucial pathways will draw us closer to CRC treatment breakthroughs.

## Figures and Tables

**Figure 4:**
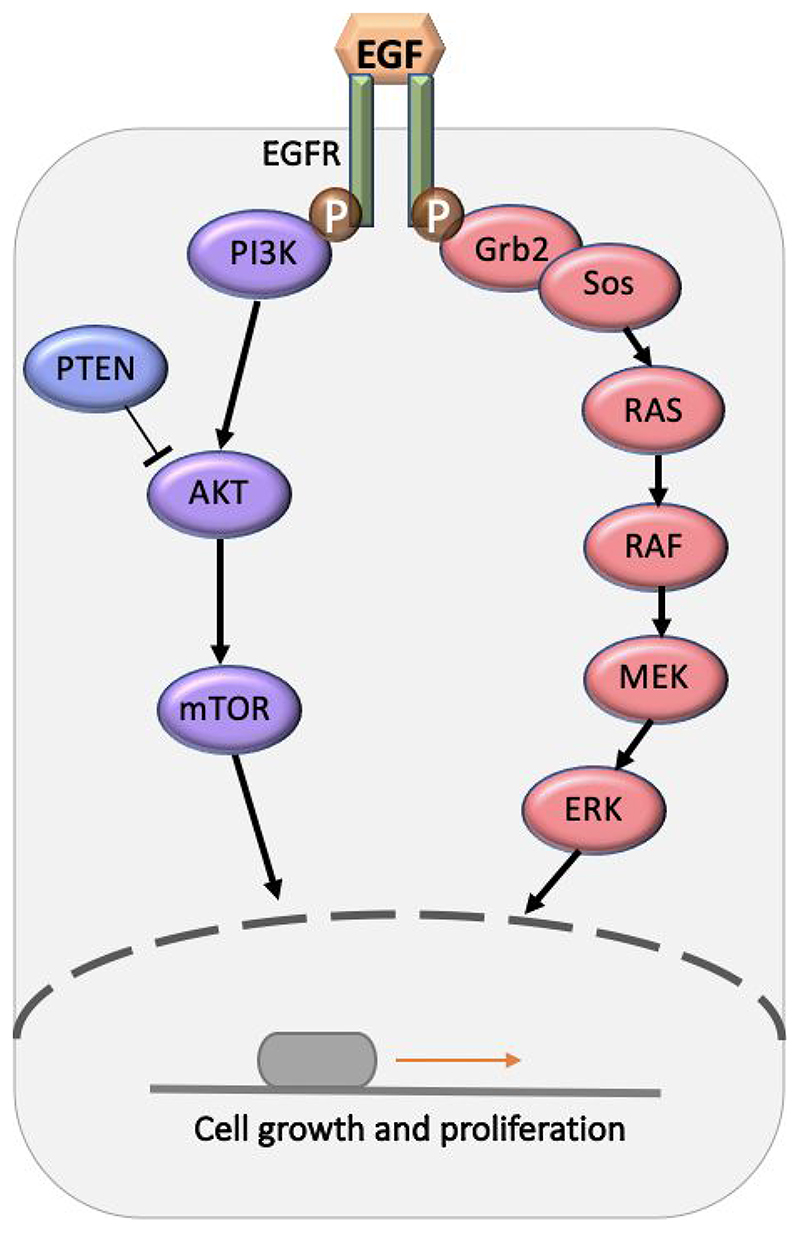
Schematic representation of the EGFR signalling pathway.
